# Study related factors associated with study engagement and student burnout among German university students

**DOI:** 10.3389/fpubh.2023.1168264

**Published:** 2023-04-20

**Authors:** Nils Olson, Renate Oberhoffer-Fritz, Barbara Reiner, Thorsten Schulz

**Affiliations:** Department of Sport and Health Sciences, Chair of Preventive Pediatrics, Technical University of Munich, Munich, Bavaria, Germany

**Keywords:** student burnout, study engagement, workload, university, student, resilience, study satisfaction, study demands-resources

## Abstract

**Introduction:**

Student burnout has become a health concern in higher education systems. Its prevalence rates are high due to specific demands in this life situation. It leads not only to increased academic dropout rates but is also associated with negative health outcomes both physically and mentally. Its counterpart is study engagement, which is a positive, fulfilling, study-related attitude characterized by energy, dedication, and absorption. There has not been a systematical approach covering the demands directly posed by the academic environment itself. Additionally, academic subject fields apart from medicine and nursing sciences have been mostly neglected in regards to this research field. The aim of the study is therefore to identify contributing factors for both burnout and engagement within the academic environment in a sample of different subject fields at a German university.

**Methods:**

In a cross-sectional study, a sample of 3,451 students of all academic subjects at a technical university in Germany has been analyzed using an online survey. Sociodemographic data, study engagement, student burnout, study satisfaction, academic workload, number of semesters and occupational liabilities have been analyzed. Binary logistic regression was used to determine the associations of burnout symptoms and study engagement.

**Results:**

Almost a third of the students showed frequent burnout symptoms, while 42.5% showed a high degree of study engagement with no differences in gender. Age was identified as a risk factor for frequent signs of cynicism (OR = 1.073). Study satisfaction (OR between 0.459 and 0.702), semester progression (OR = 0.959) and working moderately (OR between 0.605 and 0.637) was associated with fewer symptoms in different burnout-dimensions. Study satisfaction is positively associated with study engagement (OR = 2.676). Academic workload is positively related to both burnout (OR between 1.014 and 1.021) and study engagement (OR = 1.014).

**Discussion:**

A substantial number of students show frequent symptoms of burnout and the majority is not highly engaged. The included factors contribute to the model to various degrees and show that university-bound factors play a major role. Fostering a supportive environment is key for study engagement, health and well-being. The inclusion of further, individual factors should be a future concern in order to find and promote strategies for a healthy education system.

## Introduction

The quality of an education system is currently defined in terms of academic achievement and students’ well-being (1), with their occurrence being in a reciprocal relationship. Both factors are desired outcomes by educational institutions according to the Organization for Economic Co-operation and Development (OECD). But ‘well-being’ does not play a role as a factor in international university rankings, unlike performance and impact. This discrepancy often fosters the prioritizing of achievement and output over well-being and health. The focus on students’ health is more recent and has been fueled by the health concerns caused by the European Bologna reform process and the implication it has on students (2, 3). Furthermore, students are in a special stage of their lives. It is often characterized by various changes in the individual’s social environment and lifestyle, like the detachment from family and the parental home, the establishment of new social contacts and an entry into a daily working life on top of the academic tasks (4).

From occupational research, two contrastive factors have been identified that derive from occupational well-being or its absence: *work engagement* and *burnout* (5). These factors have been found to be applicable not only in the working context but also in educational systems like schools and universities. They have therefore recently become the subject of scientific research in regards to students’ health and well-being (6–12).

Burnout is defined as a state of reduced capacity for experience with concomitant emotional and physical exhaustion as well as depersonalization, the feeling of reduced coping and cognitive slowdown (13). It is characterized by a combination of *emotional exhaustion* (EE), *cynicism* (CY) and the feeling of *reduced personal accomplishment* (RPA) (14). Originally, the term burnout referred only to people, who work in the human services and in educational or health sectors and was seen as the result of prolonged emotional strain from intense engagement with people in the work environment (7). However, burnout is also occurring in higher education settings, where the term ‘student burnout’ has been established. The prevalence of burnout is high in the student population, with one fourth to one third of the students suffering from frequent exhaustion (15, 16) and often exceeding their age matched peers (17, 18). It is therefore, that the examined contributing factors for student burnout have been directed towards the study environment and academic activities. In general, the dimensions of students burnout correspond to the dimensions of work related burnout with only ‘RPA’ being reframed to ‘reduced academic efficacy’ (RAE). Student burnout has been linked to increased suicidal thoughts, lower self-esteem and high university drop-out rates to name but a few (18–21).

While the definition of burnout has already been used in the late 1960s (22) (study) engagement is a more recent construct. Study engagement in particular forms the positive antipode of student burnout. In fact, student burnout is considered as an erosion of academic engagement (7). It contributes to a positive, rewarding and fulfilling state of mind, a high energy level and positive study-related emotions. A high engagement is related to positive health outcomes (23, 24).

Even though only few studies have been conducted on study engagement in conjunction with student burnout, there are some interesting findings:

Burnout prevalence-rates of 12% to over 70% were documented among students of different majors, most of which have been medical students. Among those students who did not exhibit burnout in the cited studies, there were many individuals who were already at increased risk for its development (25–29).

With only few studies investigating the prevalence of study engagement, it can only be assumed that barely half of the students are highly engaged within their study courses (15, 30).

There is inconsistent evidence for the role of gender for engagement and burnout, but slightly more often has the female gender been linked to higher rates of burnout (27, 28, 30, 31). Likewise, there is an inconsistent evidence based tendency for females to experience more study engagement than males (30). However, other studies did not identify differences between genders (32, 33).

Apart from socio-demographic factors, influences from the academic setting as well as personal resources have also been included into some of the recent research analysis. Attempts have been made to find different protective factors and risk factors within the field of engagement and burnout, some following the Job-Demands-Resources Model (JD-R) (34). The JD-R model is a simple, resilience based model, describing the existence of risk factors (demands) and protective factors (resources) for the development of burnout and engagement in the workplace as well as in the educational field. Adapted to university life, several factors have been tested for their fit in one of the categories. These factors include study-related parameters (e.g., duration and stage of studying, study satisfaction, supervisor support, moonlighting, scope of action and academic workload) and/or personal resources and/or risk factors (e.g., health behavior, mental health status, psychological flexibility, marital status, etc.) (30, 33, 35–37). However, there is little homogeneity between the study protocols and the included parameters.

In general, study engagement in conjunction with student burnout is still an underexplored topic within the higher education systems. Only few studies with a person-oriented approach exist in this field and often those studies are limited to students of the medical fields. Other majors have been broadly neglected so far. Also, there is only little consistency between study-methods and the included resources and risk factors.

We therefore aim at investigating factors that are related to the life of students and the academic field in order to identify their contribution to student burnout and study engagement among a large sample of students from all majors at a German university. These include:

(1) Amount of semesters studied.
(2) Study satisfaction.
(3) Academic workload.
(4) Time investment for side-occupations.

We aim to answer the following research questions in this study:

(1) What are the prevalence rates of student burnout and study engagement amongst students of all majors at a German university?
(2) To what extent are socio-demographic and study related factors associated in the occurrence of both study engagement and student burnout?

This will help to deduce measures to prevent burnout and foster engagement on a long-term basis and find key components of the university-life that need to be highlighted in order to foster resilience and reduce risk factors.

## Methods

For the present cross sectional study-design an online-survey in German language was used, which was based on theoretical preliminary considerations, comprising 18 different health dimensions and—depending on the filter guide—consisted of up to 89 questions. The majority of the question blocks comprised international standardized and validated questionnaires. Additional anthropometric, socio-demographic and study-related information were included. For the present study, we used the data on age, gender, number of semesters, the Utrecht Work Engagement Scale for Students (UWES-S 9), the Maslach-Burnout-Inventory Short Form for Students (MBI-SS), data on academic workload, study satisfaction and occupational working hours.

From November 2019 to January 2020, students enrolled at the Technical University of Munich (*n* = 45,876) have been contacted *via* e-mail and asked to fill in the standardized online questionnaire. Participation was voluntary and not rewarded. Only individuals who were 18 years or older were included in the study. A total of 4,720 students (response rate = 10.3%) participated, of which 3,451 were included into this study.

### Burnout: Maslach-burnout-inventory short form for students

The MBI-SS, which was developed for the assessment of burnout within the circumstances of an academic setting, was used to assess burnout symptoms (7). It consists of three burnout dimensions: Emotional Exhaustion (EE), which can be described as fatigue caused by study demands and represents the individual stress component of this syndrome. Cynicism (CY), which can be seen as the individual’s mental distance from his or her studies or excessively detached responses to other students and teachers in the academic setting. It represents the interpersonal component of burnout. Reduced Academic Efficacy (RAE), which can be defined as a feeling of decline in one’s competence and productivity and a diminished sense of accomplishment. It represents the self-evaluation component of burnout (12, 38, 39). Each dimension was measured with three items. The frequency for each item was scored on a 7-point Likert scale and ranged from 0 (never) to 6 (always). A higher score indicates more frequent symptoms. The three-dimensional criteria were used independently, and the mean score of each dimension was used to assess the respective symptom. Groups were formed of individuals who express ‘frequent’ symptoms in each of the three burnout dimensions, defined as symptoms occurring at least once per week. The MBI-SS has been used to measure student burnout across several countries with good compliance with the quality criteria (40, 41).

### Study engagement: UWES-S 9

The Utrecht Work Engagement Scale-Student Survey (UWES-S 9) (7) measures levels of engagement and includes three dimensions: Vigor (i.e., having high levels of energy during studying), Dedication (i.e., perceiving one’s studies as important and meaningful), and Absorption (i.e., being immersed in one’s studies). The items of the UWES-S are scored similarly to those of the MBI-SS. The UWES-S has been validated internationally (7, 11, 35, 42). The mean value from the sum of all three dimensions was calculated, and values from 0 to 6 were assumed. A higher value indicating a higher engagement. Students with a value above 3.5 were grouped as ‘highly engaged’.

### Study and lifestyle related factors

We included study related factors and factors distinct for the students’ lifestyle as additional factors:

(1) *Study satisfaction*, as an indicator of how content students are with their choice of study.
(2) *Side-job work intensity*, as an objective indicator of weekly hours spent for occupational liabilities.
(3) *Amount of semesters studied*, as an indicator of the duration that a student is enrolled in a certain study program.
(4) *Academic workload*, as an indicator of the weekly hours spent for academic tasks.

Students could rate their s*tudy satisfaction* on a 10-point Likert scale, with 10 being ‘very content with the choice of study courses’ and 1 as ‘not content at all’. We measured o*ccupation* in weekly hours and grouped the answers in four categories: *No occupation*, *up to 10* working hours per week, *10–20 h* of weekly occupation and *more than 20 h*. *Study workload* was divided into ‘time spent at university’, ‘in classes’ and ‘in the preparation and revision of learning material’ both during the semester and during the lecture-free period, with the latter being the time most students write the majority of their exams and course assessments.

### Statistical analysis

All descriptive data are expressed as mean (*M*) ± standard deviation (SD) for metric variables, while frequencies of observations and their corresponding percentages are reported for categorical variables. Pearson’s correlation analysis has been performed between all metric variables.

The one-factor structure of the UWES-S 9 has been used due to a high internal correlation of its three dimension Vigor, Dedication and Absorption (0.87–0.91) (43); for burnout the three-factor structure has been maintained.

The students t-test was used to analyze differences between groups. Pearson’s correlation coefficient *r* has been indicated as the effect size. Binary regression was used to analyze predictors for the presence of ‘high’ study engagement and of ‘frequent’ burnout symptoms in each dimension. Non-Binary individuals could not be included in the regression models due to the small sample size. They were excluded from the regression analysis but not from descriptive statistics.

For all analyses, a probability value of *p* ≤ 0.05 was considered statistically significant. All analyses were performed using SPSS 29.0 (IBM Inc., Armonk, New York, NY, United States).

## Results

Only those participants were included who completed the UWES-S 9 as well as the MBI-SS, leading to an inclusion of 3,451 students of which 3,447 indicated their gender; among them 42.5% male (*n* = 1,466), 57.1% female (*n* = 1,967) and 0.4% non-binary (*n* = 14). Age ranged from 18 to 55 years and averaged at 22.5 ± 3.42 years.

Students were between the 1st and 25th semester of their current degree program, with a mean of 3.6 ± 2.62 semesters. A large proportion of participants were students in their first or second semester (*n* = 1,221, 35.6%). Third and fourth semester accounted for 29,0% (*n* = 994), fifth and sixth for 21,3% (*n* = 732), seventh to ninth for 11,1% (*n* = 381) and tenth and above for 3,0% (*n* = 103).

Students spent an average of 31.8 ± 16.93 h per week for academic activities during the semester (visiting lectures and revising and preparing material) with a maximum of 110 h and a minimum of 0 h. 37.4 ± 22.10 h were spent for those activities between semesters, with a maximum of 115 h and a minimum of 0 h. On average, participants spent 26.2 ± 16.00 h per week at the university, of which 14.2 ± 8.92 h were spend on courses. The preparation and follow-up time was 17.6 ± 14.47 h during the semester and 23.2 ± 19.82 h during the lecture-free period.

56.7% (*n* = 1,879) of the students carried out side-jobs. Among those, the average weekly working hours were 11.3 ± 7.54 h. Employed students spent significantly less time studying. This is true for the time within the semester [*t*(2677.115) = −7.486; *p* < 0.001, *n* = 3,095] as well as for the time outside of the lecture period [*t*(2276.595) = −3.304; *p* < 0.001; *n* = 2,826].

Referring to students engagement, the average score on the UWES-S 9 was 3.3 ± 1.05, with no significant differences between male and female according to the students *t*-test [*t*(3431) = 1.254, *p* = 0.210]. The majority of the students (57.5%, *n* = 1,983) were categorized as having low to medium study engagement, opposed to 42.5% (*n* = 1,468) who were highly engaged.

Regarding burnout, the mean score of the dimension of EE was 2.7 ± 1.56. For the dimensions of CY, it was 1.2 ± 1.45, and RAE 2.0 ± 1.57. 31.8% (*n* = 1,097) of all students had frequent symptoms in at least one dimension. Accordingly, 25.4% (*n* = 875) of the students frequently experienced symptoms of EE, 7.7% (*n* = 266) symptoms of CY and 15.5% (*n* = 536) had frequent symptoms of RAE. 4.3% (*n* = 147) had frequent symptoms in all three dimensions simultaneously. There has been no difference between male and female regarding EE [*t*(3,431) = −0.468; *p* = 0.640] and CY [*t*(3,431) = 0.505; *p* = 0.614], but a significant difference in the students’ *t*-test has been observed in the dimension of RAE [*t*(3,431) = −3.487; *p* < 0.001] with males showing lower values (1.9 ± 1.55; *n* = 1,466) than females (2.1 ± 1.58; *n* = 1,967). However, the effect size is very small (*r* = 0.06; Table 1). 6.7% of the students were simultaneously engaged and burned out in at least one dimension.

**Table 1:** Means of the dimensions of the Maslach-Burnout-Inventory Short Form for Students (MBI-SS) and the sum score of Utrecht Work Engagement Scale for Students (UWES-S) among the genders.

	Engagement		Exhaustion		Cynicism		Reduced academic efficacy	
	*M* ± SD	*N*	*M* ± SD	*N*	*M* ± SD	*N*	*M* ± SD	*N*
Male	3.3 ± 1.06	1,466	2.6 ± 1.57	1,466	1.2 ± 1.48	1,466	1.9 ± 1.55	1,466
Female	3.3 ± 1.03	1,967	2.7 ± 1.54	1,967	1.2 ± 1.42	1,967	2.1 ± 1.58	1,967
Non-binary	2.7 ± 1.21	14	3.4 ± 1.68	14	2.5 ± 2.07	14	3.1 ± 1.87	14

The correlation analysis showed strong interrelations within the three burnout dimensions. Cronbach’s Alpha was 0.844 for EE, 0.871 for CY and 0.772 for RAE. Study engagement had a medium to strong negative correlation to all three burnout dimensions, with CY being associated to the largest degree. Time spent for studying weekly is positively associated with study engagement. Study satisfaction is moderately to strongly negatively related to all three burnout-dimensions and strongly positively correlated to study engagement (Table 2).

**Table 2:** Correlation table for included variables (*n* = 2,829–3,451).

Variable	*M*	SD	1	2	3	4	5	6	7	8	9
1. Emotional Exhaustion	2.7	1.56									
2. Cynicism	1.2	1.45	0.52^**^								
3. Reduced academic efficacy	2.0	1.57	0.57^**^	0.55^**^							
4. Study Engagement	3.3	1.05	−0.41^**^	−0.61^**^	−0.46^**^						
5. Age [years]	22.5	3.42	−0.06^**^	0.07^**^	−0.10^**^	−0.04^*^					
6. Semester	3.0	2.62	−0.08^**^	0.02	−0.10^**^	−0.01	0.30^**^				
7. Weekly time spent for preparation and revision of courses during semester [hours]	31.8	16.93	0.23^**^	0.06^*^	0.09^**^	0.10^**^	−0.10^**^	−0.08^**^			
8. Weekly time spent for preparation and revision of courses between semesters [hours]	37.4	22.10	0.14^**^	0.04^*^	0.07^**^	0.04	0.07^**^	−0.05^**^	0.46^**^		
9. Weekly occupational obligations [hours]	6.2	7.50	−0.08^**^	0.022	−0.07^**^	−0.03	0.25^**^	0.13^**^	−0.15^**^	−0.09^**^	
10. Study satisfaction	8.0	3.00	−0.34^**^	−0.63^**^	−0.40^**^	0.66^**^	−0.06^**^	−0.03	−0.01	−0.02	−0.04^*^

^*^Significant on the level of *p* ≤ 0.05; ^**^significant on the level of *p* ≤ 0.001.

Students showed different combinations of burnout and engagement (Table 3). The smallest proportion of students (*n* = 230; 6.7%) were both engaged and burned out in at least one dimension at the same time.

**Table 3:** Different profiles of burnout and study engagement.

Profile	*N*	% of sample	% among frequent symptoms of burnout
Frequent burnout symptoms–low to medium engagement	868	25.2	79.1
Highly engaged no frequent burnout symptoms	1,238	35.9	
Highly engaged and frequent symptoms of burnout	230	6.7	21.0
Low to medium engagement–no frequent burnout symptoms	1,115	32.3	
Total	3,451	100.0	100.0

Binary logistic regression analysis was used to assess which variables influence the probability of the presence of ‘frequent’ burnout symptoms in each of the three dimensions. Multicollinearity of predictors was absent and the Hosmer–Lemeshow-test showed valued of *p* > 0.05 in each of the calculations. The semester progression was identified as a predictor of EE with students from higher semesters being less likely to suffer from frequent symptoms (OR = 0.959). Time spent for studying during the semester had a positive directed influence on EE (OR = 1.021). Study satisfaction on the other hand had a negative influence on the occurrence of frequent EE symptoms (OR = 0.702). Occupation has also been identified as a predictor, where 10–20 working hours per week have a negative effect on the probability for frequent EE symptoms comparing to no occupation (OR = 0.605; *χ*^2^(9) = 303.958; *p* < 0.001; *n* = 2,615; Table 4).

**Table 4:** Binary regression model for emotional exhaustion.

Predictors		*B*	Odds ratio	CI 95%		*p* value	Nagelkerkes *R*^2^	*N*
						<0.001	0.164	2.615
Gender	Male–female	−0.004	0.998	0.821	1.21	0.968		
Age		−0.015	0.985	0.955	1.017	0.356		
Semester		−0.042	0.959	0.920	0.999	0.042^*^		
Study satisfaction		−0.354	0.702	0.667	0.739	<0.001^*^		
Hours spent for studies during semester		0.021	1.021	1.015	1.028	<0.001^*^		
Hours spent for studies between semesters		0.004	1.005	1.000	1.009	0.076		
Occupation						<0.001^*^		
	No occupation – up to 10 h/w	−0.201	0.824	0.647	1.033	0.092		
	No occupation −10–20 h/w	−0.449	0.605	0.465	0.792	<0.001^*^		
	No occupation – 20 h+/w	0.158	1.182	0.809	1.695	0.403		

^*^Significant on the level of *p* ≤ 0.05.

Age (OR = 1.071), study satisfaction (OR = 0.459) and academic workload during the semester (OR = 1.013) were associated to frequent CY symptoms (*χ*^2^(9) = 465.008; *p* < 0.001; *n* = 2,615; Table 5).

**Table 5:** Binary regression model for cynicism.

Predictors		*B*	Odds ratio	CI 95%		*p* value	Nagelkerkes *R*^2^	*N*
						<0.001	0.390	2,615
Gender	Male–female	−0.174	0.840	0.593	1.190	0.327		
Age		0.069	1.071	1.024	1.121	0.003^*^		
Semester		−0.007	0.993	0.928	1.061	0.827		
Study satisfaction		−0.779	0.459	0.420	0.500	<0.001^*^		
Hours spent for studies during semester		0.013	1.013	1.002	1.024	0.017^*^		
Hours spent for studies between semesters		0.004	1.004	0.995	1.012	0.390		
Occupation						0.276		
	No occupation—up to 10 h/w	−0.416	0.660	0.420	1.034	0.070		
	No occupation—10–20 h/w	−0.290	0.748	0.477	1.173	0.206		
	No occupation—20 h+/w	−0.090	0.914	0.489	1.709	0.779		

^*^Significant on the level of *p* ≤ 0.05.

The number of semesters studied (OR = 0.890) and the overall academic satisfaction (OR = 0.652) as well as 10–20 h of weekly occupation compared to no occupation (OR = 0.638) predict frequent symptoms of RAE (*χ*^2^(9) = 257.178; *p* < 0.001; *n* = 2,615; Table 6).

**Table 6:** Binary regression model for reduced academic efficacy.

Predictors		*B*	Odds ratio	CI 95%		*p* value	Nagelkerkes *R*^2^	*N*
						<0.001^*^	0.167	2,615
Gender	Male–female	0.126	1.135	0.896	1.437	0.295		
Age		−0.019	0.981	0.945	1.019	0.327		
Semester		−0.116	0.890	0.844	0.940	<0.001^*^		
Study satisfaction		−0.428	0.652	0.615	0.691	<0.001^*^		
Hours spent for studies during semester		0.007	1.007	0.999	1.014	0.086		
Hours spent for studies between semesters		0.002	1.002	0.996	1.007	0.603		
Occupation						0.046^*^		
	No occupation—up to 10 h/w	−0.233	0.792	0.595	1.055	0.111		
	No occupation—10-20 h/w	−0.449	0.638	0.463	0.881	0.006^*^		
	No occupation—20 h+/w	−0.174	0.840	0.529	1.335	0.461		

^*^Significant on the level of *p* ≤ 0.05.

The binary logistic regression has also been used to investigate the influence of the variables on the study engagement. The overall academic satisfaction (OR = 2.670) and the amount of hours spent for academic tasks during the semester (OR = 1.013) have been identified as predictors for being highly engaged (*χ*^2^(9) = 895,729; *p* < 0.001; *n* = 2,615; Table 7).

**Table 7:** Binary regression model for study engagement.

Predictors		*B*	Odds ratio	CI 95%		*p* value	Nagelkerkes *R*^2^	*N*
						<0.001^*^	0.389	2,615
Gender	Male–female	−0.015	0.985	0.818	1.188	0.877		
Age		0.014	1.014	0.983	1.046	0.393		
Semester		0.019	1.019	0.980	1.059	0.344		
Study satisfaction		0.982	2.670	2.450	2.910	<0.001^*^		
Hours spent for studies during semester		0.013	1.013	1.007	1.020	<0.001^*^		
Hours spent for studies between semesters		−0.002	0.998	0.994	1.003	0.532		
Occupation						0.827		
	No occupation—up to 10 h/w	−0.038	0.963	0.767	1.209	0.775		
	No occupation—10-20 h/w	−0.107	0.898	0.703	1.148	0.391		
	No occupation—20 h+/w	−0.119	0.888	0.603	1.307	0.548		

^*^Significant on the level of *p* ≤ 0.05.

## Discussion

With this study, the prevalence of student burnout and study engagement among a large sample of students of all academic subject fields at a German university was undertaken. Associations between socio-demographic and study related factors have been taken into account to find associations of study engagement and student burnout within the academic setting.

31.8% of the students showed frequent burnout symptoms (defined as at least once per week) in at least one dimension of student burnout. 25.4% of the students experienced frequent EE, 7.7% CY and 15.5% RAE. The numbers for EE and CY are relatively smaller than those from other German universities, where EE exceeded 30% and CY exceeded 25%. RAE on the other hand occurs more frequently in our sample while the respective studies only report 3.6 and 6.1% of affected students (44). In almost all of the cases of any frequent burnout symptoms in our study, frequent EE was present and almost half of the respective students showed symptoms exclusively in EE. This may be attributed to the fact that EE is described as the initial burnout symptom, which naturally occurs first. With increasing time of persistence, this can lead to additional symptoms within the other dimensions (45, 46).

Burnout has originally been described as the antipode of engagement, with burnout being the erosion of engagement (7). We found however, that a small but substantial number of students (6.7%) are simultaneously engaged and show frequent symptoms of burnout in at least one dimension. Salmela-Aro and Read (30) also identified profiles of students with high engagement and concomitant burnout symptoms. This poses the question how accurate the underlying framework of the relationship between burnout and engagement really is. Nevertheless, our results have to be considered in the light of being subclinical self-assessments.

42.5% of the study population were highly engaged. This is slightly less than has been shown in a German-wide investigation in 2017, in which 47.5% of the students displayed a high study engagement (15). Our analysis did not find any differences between men and women. This adds to the body of evidence, which has to this day produced inconsistent findings regarding the role of sex in study engagement. 79.1% of the students, who express frequent burnout symptoms, were low to moderately engaged, while the highly engaged students mostly did not display burnout symptoms. Engagement as a protective trait in regards to burnout seems at least plausible.

There has been no significant difference when comparing the mean scores of EE and CY between males and females. And even though there has been a significant difference in the t-test when examining RAE, we do not consider it to be relevant taking the very small effect size of *r* = 0.06 and the big sample size into account. Also, when analyzing for any gender associations within the binary logistic regression, we found no difference in the likelihood of the presence of frequent symptoms in any burnout dimension, so we conclude no differences between the sexes when it comes to burnout.

We found that the hours spent for academic tasks during the semester and between semesters is attributing to the occurrence of frequent symptoms of EE. Each additional hour spent for academic tasks increases the risk for frequent EE symptoms by 2.1%. CY is also associated with a higher time investment within the semester with each additional hour increasing the likelihood by 1.4%. RAE symptoms seem to be unaffected by hours of study involvement. Time expenditure has been linked to burnout before (33, 36, 47, 48). However, subjective workload (e.g., ‘too much work to do’) has been postulated as a better indicator for burnout than objective measures (49), we still find significant results when comparing objective numbers, in due consideration of the self-reported methodology. The fact that there is a difference between the influences of the academic workload during and between semesters might explain that some studies did not produce significant results when investigating the overall, objective academic workload.

It is notable that the study engagement score is also affected by workload but it manifests as a resource rather than a demand. Each additional hour of academic work increases the probability for high study engagement by 1.4%. It has indeed been shown that a higher engagement can lead to a higher time investment (50). We conclude that the triangulated relationship of engagement, study workload, and burnout is contextual. More hours spent on studying can be a cause of burnout but also an indicator of high engagement, thus presenting itself as a resource or a burden.

Having a side-occupation had interesting associations with EE and RAE. First, it has been shown, that students, who have a paid job, spend less time studying. Nevertheless, when controlling for this factor within the regression analysis, it still showed that working for 10–20 h a week decreased the likelihood for frequent symptoms to almost half for EE and more than half for RAE. Working less than 10 h or more than 20 h does not seem to affect the likelihood for either burnout dimension. A more linear relationship would seem more intuitive, which is not the case in our sample. Maybe activities outside of the academic field can contribute to distraction, provide a better financial situation (51) or teach students to take responsibilities, which could all have a beneficial effect on the perception of academic strains. Earlier studies failed to find significant connections between side-occupation and stress (52) maybe the non-linear relationship is a cause for that. In a study with US medical residents side-occupation was even found to grant benefits for the overall quality of life and the work-life-balance (53).

Study satisfaction was found to influence burnout and study engagement in a healthy manner in our sample. In occupational research the role of job satisfaction has been shown to positively correlate with engagement (52) and in the setting of university, study dissatisfaction has been identified as a predictor of burnout (54). As ‘satisfaction’ was not further specified in this study’s questionnaire, it can relate to several and also different aspects of studying for each of the participants. Satisfaction with supervisor support is one of the constructs that has been linked to an increase in study engagement before (33), which certainly contributes to the overall study satisfaction. In future studies, contributing factors to study satisfaction should be elaborated more as it seems to be an important factor in regards to engagement and burnout.

It has been postulated, based on previous research, that with increasing number of semesters and age, stress and burnout symptoms – especially symptoms of CY and RAE – are increasing (55, 56). This stems from the assumption that academic problems and the resulting delay in progression lead to stress and the feeling of inadequacy. We found this to be true for CY, as the chances of suffering from frequent symptoms increased by 7.3% for each additional year of age. However, the number of semester is negatively associated with EE and RAE, with a 4.1 and 11.1% respective decrease in chances of occurrence with each increasing semester. This discrepancy between our results and the literature might stem from the fact, that duration of studying does not equal delay. A positive experience of studying that derives from a successful study-progression and the concomitant feeling of accomplishment can increase self-efficacy instead of undermining it. The mere number of semesters might not be an adequate indicator for the risk factor that we postulated it to be. For future studies, there should be a distinction between study progress and unintentional delay.

## Conclusion

Burnout is a major problem in higher education setting, with almost one third of the students being affected. We could not identify any relevant differences between gender. We found that study engagement and student burnout can be predicted by some of the included variables. In this process, we identified study satisfaction as a main variables for both burnout and engagement. Academic workload has been identified as being more complex as to put it in one of the categories ‘risk factor’ or ‘protective factor’ as it seems to manifest as both: it is possible that more hours spent for studying can be a cause for burnout but also a ‘symptom’ of high engagement. However, the temporal and causal relationship of the identified factors is not clear and needs to be confirmed by longitudinal research or intervention studies.

The results of this survey also help to further develop models like the initially mentioned JD-R model. We have found that there are protective factors from burnout, which contradicts the original structure of the model, where resources effect engagement only and demands effect burnout only. These cross-effects have also been demonstrated by Gusy et al. (36). Also, we have found additional parameters that have to this day not been included into the model, like side-occupation and objective workload.

## Implication

Study satisfaction can only be met if the expectations and needs of students are known, which should be a concern of higher education systems aiming for a healthy students’ well-being.

Educators and institutions need to be taught about student burnout und study engagement. Students should be made aware of early signs of burnout, which should also be addressed by the university’s support systems. Eliminating all stressors is not a feasible approach but aiming for a supportive environment, fostering stress management and strengthening individual resources by courses in these fields may significantly affect burnout and engagement among students.

The study showed that the whole student population is at increased risk for burnout and not only students of medical subjects, as often displayed in past research. Further studies are needed to gain insight about different academic subject groups.

The inclusion of further, individual factors from the preventive fields of nutrition, physical activity, stress management and substance abuse should be systematically integrated into the research topic in order to better individualize and specify the health promotion measures in the sense of behavioral and situational prevention at the universities.

## Data availability statement

The raw data supporting the conclusions of this article will be made available by the authors, without undue reservation. Other data related to this study is not publicly available because it is included within upcoming articles and cannot be shared at this time.

## Ethics statement

The studies involving human participants were reviewed and approved by Ethics Committee of the Technical University of Munich. The patients/participants provided their written informed consent to participate in this study.

## Author contributions

RO-F, TS, BR, and NO substantially contributed to the conception and design of the research, and commented on the previous versions of the manuscript. NO, BR, and TS performed the material preparation and data collection. NO analyzed the data, interpreted the results and wrote the first draft of the manuscript. All authors contributed to the article and approved the submitted version.

## Funding

The project was funded by an unrestricted grant by the Techniker Krankenkasse.

## Conflict of interest

The authors declare that the research was conducted in the absence of any commercial or financial relationships that could be construed as a potential conflict of interest.

## Publisher’s note

All claims expressed in this article are solely those of the authors and do not necessarily represent those of their affiliated organizations, or those of the publisher, the editors and the reviewers. Any product that may be evaluated in this article, or claim that may be made by its manufacturer, is not guaranteed or endorsed by the publisher.
